# Role of miRNA in the Transmission of Metabolic Diseases Associated With Paternal Diet-Induced Obesity

**DOI:** 10.3389/fgene.2019.00337

**Published:** 2019-04-18

**Authors:** Charlotte Dupont, Laurent Kappeler, Sarah Saget, Valérie Grandjean, Rachel Lévy

**Affiliations:** ^1^ Sorbonne Université, Inserm, Centre de Recherche St-Antoine, CRSA, AP-HP, Hôpital Tenon, Service de biologie de la reproduction-CECOS, Paris, France; ^2^ Sorbonne Université, INSERM, Institute of Cardiometabolism, Centre de Recherche St-Antoine, CRSA, Paris, France; ^3^ Inserm U1065, Team Control of Gene Expression (10), Université Cote d’Azur, Nice, France

**Keywords:** miRNA, intergenerational, epigenetics, Developmental Origins of Health and Diseases, Paternal Origins of Health and Diseases, metabolic disorders, paternal transmission

## Abstract

The concept of Developmental Origins of Health and Diseases (DOHaD) recognizes that an unfavorable maternal environment alters the developmental trajectory of the fetus and can lead to long-term risk of developing chronic noncommunicable diseases. More recently, the concept of a paternal transmission [Paternal Origins of Health and Diseases (POHaD)] has emerged stressing the impact of paternal overweight or obesity on offspring’s health and development. While very few examples of paternal epigenetic inheritance of metabolic disorders have been evidenced in human, many experimental mouse models based on high-fat diet (HFD)-induced paternal obesity have been developed to breakdown molecular mechanisms involved in the process. Besides DNA methylation and chromatin structure, sperm short noncoding RNAs have been considered as the main epigenetic vector of inheritance of paternally environmentally induced changes. Among them, sperm miRNAs are one particular subspecies sensitive to environmental changes and obesity can modify the sperm miRNA profile. Once delivered into the zygote, these molecules might induce epigenetic modifications in the embryo, thereby leading to consequences for fetus development and offspring physical and metabolic health later on in life. Furthermore, some data also suggest that metabolic pathologies may be intergenerationally or transgenerationally transmitted.

## Introduction

In the 1990s, Barker’s epidemiological studies have highlighted a predisposition of metabolic syndrome in adulthood in low birth-weight individuals. This “Barker hypothesis” has been the starting point of many works (Hales and Barker, 1992; Barker et al., 2002). The concept of maternal nutritional footprint has emerged and has extended toward the concept of Developmental Origins of Health and Diseases (DOHaD) (Gluckman and Hanson, 2004). This concept recognizes that maternal environment can alter the trajectory of fetal development through various mechanisms such as hormonal imbalance, oxidative stress, and epigenetic changes. Organs development and function may be affected, thereby leading to a long-term increased risk of developing several pathologies such as metabolic syndrome, cardiovascular diseases, autoimmune diseases, neurodegenerative diseases, cancers, and infertility (Eriksson, 2016).

More recently, the concept of paternal transmission of environmental messages (POHaD: Paternal Origins of Health and Diseases) has emerged (Soubry, 2018). In humans, epidemiological studies in Sweden have indicated that paternal grandparents’ exposure to famine may predispose subsequent generations to obesity and cardiovascular diseases (Kaati et al., 2002, 2007). More recently, it was shown that paternal grandfather’s nutrition in prepuberty may predict grandchildren’s mortality from cancer (Vagero et al., 2018). This phenomenon has been more widely studied in rodent models, although numerous clinical studies are in progress. Indeed, male mice offspring of fathers that fed a low-protein diet presented lower liver cholesterol esters and impaired liver gene expression involved in the biosynthesis of lipids and cholesterol and changes in cytosine methylation at several loci (Carone et al., 2010). In a paternal model of high-fat diet (HFD), metabolic disorders and alteration of β-cell function have been observed in adult female rats’ offspring (Ng et al., 2010). Pancreatic islet and retroperitoneal white adipose tissue transcriptomes were both altered. Expression of genes involved in aging and noncommunicable chronic disorders was particularly affected (Ng et al., 2014). Separately, in a mouse model of HFD-induced obesity, it was reported that male and female offspring developed glucose intolerance, insulin resistance, and obesity as well as impaired reproductive functions in both male and female, F1 and F2 offspring (Fullston et al., 2013). However, the phenotypes were differentially expressed according to a sexual dimorphism: obesity and glucose intolerance were observed in both F1 males and females, but only in the F2 males from the F1 maternal line. Insulin resistance was found in all animals except the F2 males from the F1 male line. Gametes quantity and quality were affected in both F1 and F2, male and female (Fullston et al., 2013).

As indicated above, environment may affect several subsequent generations. Consequently, it is important to define the terms “transgenerational” and “intergenerational.” During pregnancy or gestation, the maternal (F0) environment can modulate the development of the fetus (F1), which can have long-term effects. Importantly, this may involve development of all fetal tissues including the germ cells in reproductive ones, which may directly affect the second (F2) generation. This is called multigenerational or intergenerational transmission. In the case of transmission to subsequent generations (F3 and above), the transmission is called transgenerational because there is no direct contact between the environment and the individual’s cells. If the exposure is paternal, intergenerational transmission is limited to the F1 generation that has been exposed by paternal gametes to the environment. For subsequent generations (F2 and above), we speak of transgenerational transmission (Skinner, 2010; Heard and Martienssen, 2014; Miska and Ferguson-Smith, 2016). With the concept of intergenerational and transgenerational programming, the notion of sexual dimorphism has broadened. Indeed, males and females present strong differences in regard to their susceptibility to unfavorable programming. Sexual dimorphism is observed according to the sex of the transmitting parent, the individual, and the generation (Gabory et al., 2009).

## The Role of Epigenetic Mechanisms

Mechanisms involved in the DOHaD and POHaD phenomena are numerous, complex, and not completely elucidated. In animal models and in humans, it is known that an unfavorable pre-conceptional paternal environment such as obesity can alter conventional sperm parameters such as sperm concentration and motility (Sermondade et al., 2013; Raad et al., 2019). Importantly, it also alters the quality of gametes and molecular parameter, notably by modifying the integrity of sperm DNA (Dupont et al., 2013) and epigenetic marks (Grandjean et al., 2015). These modifications potentially alter the developmental trajectory of the future embryo (Raad et al., 2019), and consequences of paternal obesity can thus be noted very early in offspring development. Indeed, it was observed that paternal obesity is associated with altered preimplantation embryo development, notably a modified kinetics of cell division and a decreased blastocyst cell number (Bakos et al., 2011; Binder et al., 2015; Schliep et al., 2015).

Epigenetics describes processes of complex modifications of gene expression without changes to the DNA sequence. It is a crucial mechanism for cell specialization and fetus development and serves as an interface between the environment and cells. Moreover, epigenetic mechanisms have important roles in environmental adaptations and may then predict future health of the fetus (Day et al., 2016). The main mechanisms accounting for the epigenetic regulation of gene expression are DNA methylation, posttranslational histone modifications, and small noncoding RNA.

The epigenetic modulation of the intergenerational impacts of paternal obesity on offspring health and development is increasingly well documented (Lane et al., 2014). In humans, paternal obesity has been associated with changes in DNA methylation of offspring’s cord blood cells. Demethylation in the IGF2 region (Soubry et al., 2013) and in other genes involved in metabolic regulation, such as MEST, PEG3, and NNAT, has been observed (Soubry et al., 2015). Moreover, paternal obesity has also been associated with changes in gene expression and global DNA methylation of extraembryonic tissue. Peroxisome proliferator-activated receptors (PPARs) and CASP12 expression were significantly downregulated in male placentas. In female placentas, significant increase in total DNA methylation was observed (Binder et al., 2015).

Sperm epigenetic modifications may play a critical role in the mechanism of inheritance of environmentally induced changes. The role of DNA methylation in paternal transmission has been widely assessed in animals, and several studies have highlighted DNA methylation modifications in case of paternal transmission of diseases. Indeed, de Castro Barbosa et al. have observed a reprogramming of sperm DNA methylation profile in male rats fed with a HFD. Eighteen regions were found differentially methylated, and 92 genes related to DMRs were found commonly regulated by HFD in both F0 and F1 spermatozoa (de Castro Barbosa et al., 2016). Likewise, it was observed that spermatozoa produced by the obese mice fed with a HFD were hypomethylated (Fullston et al., 2013). Finally, most of the studies show that a small number of sperm loci present differential methylation according to paternal feeding. However, only a limited subset is maintained in the offspring tissues (Sharma and Rando, 2017). Consequently, whether or not DNA methylation is involved in epigenetic inheritance is still debated (Shea et al., 2015). Indeed, sperm DNA is strongly demethylated after fertilization in the oocyte, although imprinted genes escape reprogramming, and undergoes progressive re-methylation in the embryo (Smallwood and Kelsey, 2012). But some genes are more likely to respond to environmental stress, and some have been recently identified (Kazachenka et al., 2018).

In view of the specific chromatin conformation and notably the replacement of histones by protamines and the compaction in spermatozoa, posttranslational histone modifications were not widely studied. Nevertheless, small amounts of histone are not replaced by protamines during spermatogenesis, thereby suggesting their potential role in paternal transmission of noncommunicable diseases (Yoshida et al., 2018). Thus, by overexpressing the human KDM1A histone lysine 4 demethylase during mouse spermatogenesis, a team has highlighted the importance of correct histone methylation during spermatogenesis for offspring development (Siklenka et al., 2015). Furthermore, it was shown in a *Drosophila* model of paternal-diet-induced intergenerational metabolic reprogramming the importance of sperm chromatin state (Ost et al., 2014).

**Figure 1:**
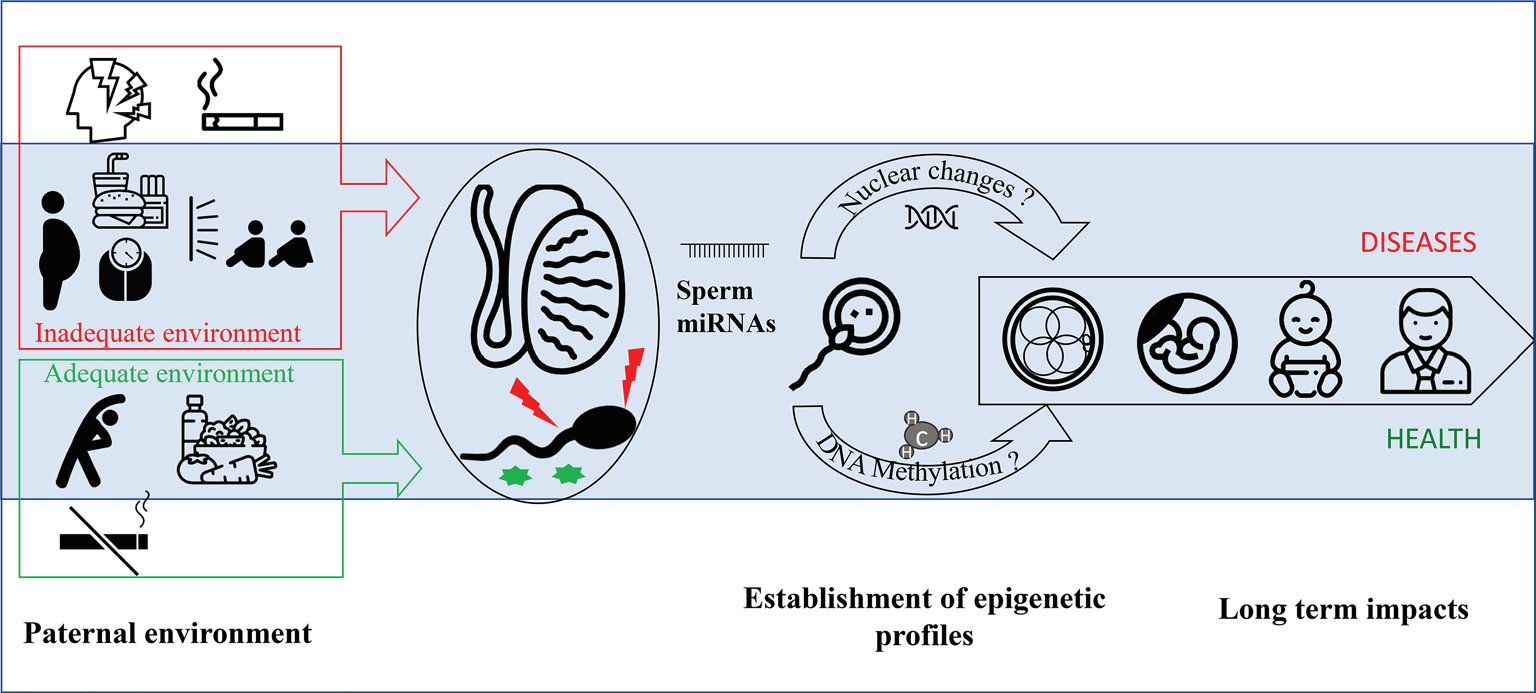
miRNAs are implicated in the transmission of paternal environmental and lifestyle conditions to offspring. Paternal environmental and lifestyle factors impact sperm quality and epigenetic profile. In contrast to healthy behavior (i.e., balanced diet, low stress, no smoking, and regular exercise), obesity and exposure to environmental pollutants including smoking have been associated with increased sperm oxidative damage, decreased vitality, and poorer DNA integrity. The unfavorable paternal factors have been suggested to alter the expression levels of various miRNAs in testis, spermatozoa, and seminal fluid. While miRNAs in testis may modulate spermatogenesis, those in spermatozoa and seminal fluid may ultimately influence early fetal development through interactions with the endometrial environment. Collectively, the mounting evidence indicates that miRNAs are epigenetic vectors of inheritance for paternal environmental conditions to influence the risk for metabolic pathologies in offspring.

By contrast, there is greater evidence that RNA molecules are strong epigenetic vectors of paternally mediated inheritance of environmental exposures. Indeed, some studies indicate that male intergenerational epigenetic signals may be transmitted by the sperm itself and/or by seminal fluid (Rando, 2012; Gannon et al., 2014), which contain a high concentration of small RNA (Figure 1). Indeed, some data indicate that seminal plasma alone may influence offspring development, as it was observed in a model of mice offspring conceived by artificial insemination associated to a mating with vasectomized male fed a low-protein diet (Watkins et al., 2018).

## Implication of Small RNAs

Male germ cells are rich in small noncoding RNAs (sncRNAs) such as micro-RNA (miRNA), endogenous small interfering RNAs (endo-siRNAs), piwi-interacting RNAs (piRNAs), and transfer RNAs (tRNAs). sncRNAs do not encode functional proteins, and according to their type, their length varies; miRNAs are about 22 nucleotides in length, endo-siRNAs are 21 nucleotides in length, and piRNAs are approximately 26–31 nucleotides in length (Song et al., 2011). tsRNAs can be divided into two main types: tRNA-derived stress-induced RNA (tiRNAs) with 28–36 nucleotides length and tRNA-derived fragment (trfRNAs) with 14–30 nucleotides length (Li et al., 2018). tRNA-derived small RNAs (tsRNAs) derive from tRNA and have attracted great attention as they can have many regulatory roles (Li et al., 2018). Thus, a difference in expression level of specific miRNAs, piRNAs, and tRNA fragments between lean and obese men spermatozoa has been observed. The authors have suggested that the altered piRNAs expression may contribute to offspring predisposition to obesity (Donkin et al., 2016). More importantly, injection of tsRNA, isolated from spermatozoa collected from HFD male, into wild-type zygotes, led to metabolic disorders in the F1 offspring. Altered expression of genes involved in metabolic pathways was observed in the early embryos and islets of F1 offspring (Chen et al., 2016). Finally, it was, also, observed in mice sperm that tRNA-gly and tRNA-Pro were both altered by running. Moreover, in this study, paternal exercise was associated with modified behavioral phenotype in offspring (Short et al., 2017).

Among the small noncoding RNAs, the microRNAs (miRNAs) are by far the most studied ones and we focus our review on them. miRNAs derive from long primary transcripts (pri-miRNA) and are modified in the nucleus by RNAse III (Drosha) to form precursors (pre-miRNA). After the nucleocytoplasmic translocation *via* exportin 5, the pre-miRNAs are modified by the enzyme Dicer into mature miRNAs. miRNA regulates gene expression at a posttranscriptional level by inhibiting the translation of their respective target genes by homologous binding to RNA (Bartel, 2004). They regulate more than 60% of the genes encoding proteins (Friedman et al., 2009). miRNAs are involved in almost all cellular functions, and in particular, proliferation, differentiation, and apoptosis (Bueno and Malumbres, 2011). They play a critical role in the pathogenesis of diseases such as cancer, cardiovascular diseases, endocrine dysfunctions, and infectious illness (Lekka and Hall, 2018).

## miRNA and Male Reproductive Functions

miRNAs are abundantly present in spermatozoa and seminal plasma. Alteration of miRNA profile may challenge spermatogenesis and sperm maturation and may be involved in male fertility (Gunes et al., 2016). Indeed, it has been observed that some miRNAs, such as miR-17-92 cluster and miR-290-295 cluster, are highly expressed in primordial germ cells and spermatogonia. The crucial role of miRNAs in spermatogenesis has been shown in Dicer knockout mice that presented altered spermatogenesis (Hayashi et al., 2008). miRNAs modulate the onset of primordial germ cell and somatic cell differentiation during mouse embryonic gonadal development (Miska and Ferguson-Smith, 2016). More recently, it was demonstrated that miRNAs are involved in gonadal sexual differentiation in both primordial germ cells and gonadal somatic cells (Fernandez-Perez et al., 2018).

Moreover, miRNAs are differentially expressed through the epididymis regions, thus playing a critical role in regulation of gene expression involved in epididymis functions (Browne et al., 2018). The sperm miRNA profile is modified along the journey through the epididymis, and epididymosomes that contain more than 350 miRNAs that play a critical role in this phenomenon (Reilly et al., 2016). Some miRNA profiles were established in testis, spermatozoa, and seminal plasma. Numerous miRNAs have been found downregulated or upregulated in azoospermic or oligozoospermic patients (Gunes et al., 2016). It has been difficult to highlight one miRNA or a certain miRNA profile that could precisely identify impairment of the male reproductive system. However, certain miRNAs have been consistently observed to be downregulated in case of spermatogenic defects (Gunes et al., 2016). Indeed, miR-34b, miR-34c, and miR-19a have been observed downregulated in testicular tissues, spermatozoa, or seminal plasma in case of oligozoospermia or azoospermia (Lian et al., 2009; Wang et al., 2011; Abu-Halima et al., 2013, 2014). Some miRNAs have been also correlated with spermatic parameters and sperm DNA integrity. Thus, three miRNAs were regularly found downregulated in case of sperm alteration (miR34c-5p, miR-122, and miR-34b-5p), and miR-429 was regularly found upregulated (Abu-Halima et al., 2013, 2014; Salas-Huetos et al., 2014, 2016). These miRNAs could represent new biomarkers of fertility, but due to the variety of miRNAs that exist, further validation is required to develop more accurate profiling panels (Gunes et al., 2016).

## Role of miRNAs in Intergenerational or Transgenerational Programming

Sperm miRNA profiles are very sensitive to paternal body weight and dietary factors changes. Thus, it was observed by RNA-seq analysis that obese men have increased expression of miR-155 and miR-122 in plasma and sperm compared to control (Lopez et al., 2018).

In the mouse model with HFD-induced obesity, 13 miRNAs were found differentially expressed in spermatozoa by microRNA array. The authors have hypothesized that these miRNAs may be responsible for paternal transmission of metabolic and reproductive phenotype (Fullston et al., 2016). In the same way, F1, F2, and F3 offspring of obese prediabetic male rats were assessed for metabolic functions. Glucose and lipid metabolism defects were observed in the three generations, with the phenotype in the F3 largely attenuated. The authors have highlighted that sperm miRNAs could be involved in the mechanism of inheritance, since 15 miRNAs were observed upregulated and 9 miRNAs downregulated, by RNA-seq analysis, in F1 male from obese fathers (Cropley et al., 2016). Offspring and the second generation (F2) from male rats fed with a HFD were lighter at birth and had a decrease of pancreatic beta-cell mass, and the phenotype was also inherited in the F2 (Cropley et al., 2016). Importantly, epigenome (DNA methylation and miRNA profiles) of F0 and F1 HFD-founders’ sperm was modified. Using RNA-seq analysis, the authors found 15 miRNAs differentially expressed in HFD-rat sperms compared to control. In particular, the miRNA let-7c-5p was differentially expressed in both F0 and F1 sperms. When assessed in metabolic tissues [liver, gonadal white adipose tissue, and extensor digitorum longus (EDL) muscle], let-7c appeared to be altered in HFD-fathers’ offspring. let-7 miRNA family plays a role in the control of lipid and glucose metabolism and insulin signaling pathways (de Castro Barbosa et al., 2016). These results suggest strongly that sperm miRNA might be involved in intergenerational and transgenerational inheritance.

Independent of the first publications about nongenetic paternal transmission of diseases (Carone et al., 2010; Ng et al., 2010, 2014), it was shown that modification of the zygote’s miRNA pool could lead to long-term phenotype modifications or pathologies in offspring. Thus, it was observed that injection of miR-1 in fertilized mouse eggs led to higher expression of miR-1 targeted RNA with clinical cardiac consequences (i.e., cardiac hypertrophy). miR-1 was observed abundant in sperm, giving first evidence suggesting a nongenetic transmission of paternal pathologies (Wagner et al., 2008). In another study, the injection of miR-124 (highly expressed in the brain and involved in central nervous system development) led to large body size associated with upregulated miR-124-targeted-transcripts like Sox9 (Grandjean et al., 2009). Disorders were observed from the embryo stage, as duplication of inner cell mass resulted in twin pregnancies. These studies provide first evidence of the role of paternal miRNA in fetal programming. Later, the relationship between sperm miRNA and their role in programming zygotes became clear. Indeed, it was observed that injection of sperm RNA collected from obese male mice in normal healthy zygotes led to the same metabolic disorders observed in obese fathers’ offspring. By small RNA-seq analysis, it was found 22 deregulated miRNA in male mice fed with an obesogenic diet, and direct injection of miR-19b induced similar metabolic disorders in offspring (Grandjean et al., 2015).

On the other hand, it was observed in nonobese mice that long-term physical activity alters the offspring metabolic status. The offspring of father exposed to physical activity exhibited increasing body weight and higher adiposity, glucose intolerance, and insulin resistance when exposed to HFD compared to control. Fathers exposed to physical intervention presented differential methylation in sperm DNA for some genes and altered spermatic miRNA profile. Thus by RNA-seq analysis, miR-483-3p, miR431, and miR-21 were found upregulated in spermatozoa from exercised male mice, while miR-221 was found downregulated. The authors have hypothesized a higher susceptibility to HFD-induced metabolic diseases in the offspring of fathers that exercised (Murashov et al., 2016). A balance between diet and physical activity is therefore essential.

Finally, miRNA expression is very variable and can change quickly according to the environment. Consequently, it is difficult to find a profile for a specific phenotype. Nevertheless, it may be this specificity that plays a role in the adaptation of tissues and individuals to the environment.

## Potential Pathways Involved in miRNA-Mediated Epigenetic Inheritance

Sperm miRNAs are transferred into oocyte cytoplasm during fertilization and can modulate gene expression during embryo development. miRNAs are crucial for early embryo development. Indeed, sperm miRNA deficiency, induced by Dicer and Drosha conditional knockout (cKO) in mice, impaired preimplantation developmental potential, which can be reversed by injection of RNA or small RNA (Yuan et al., 2016). Also, the blastocyst formation rate and the live birth rate of embryos resulting from intracytoplasmic sperm injection (ICSI) using sperm depleted of 90% of its content of RNA after RNAse treatment were significantly decreased (Guo et al., 2017). On the other hand, it has been shown that sperm miRNAs modulate zygotic genome activation and might play a critical role in regulating maternal miRNA turnover during zygote first cleavage (Yuan et al., 2016). In this regard, paternal miR-34c has been shown to be essential for the first embryo cleavage (Liu et al., 2012). Furthermore, we hypothesized that miRNAs impact the development of placenta, as it was observed with DNA methylation (Mitchell et al., 2017).

Pathways by which miRNAs subsequently modulate the development of individuals are not well known. Grandjean et al. have hypothesized that sperm miRNAs first act as gene modulators, but that the maintenance of the phenotype could be caused by modification of the chromatin structure, modulating then gene expression (Grandjean et al., 2009). It should be also noted that other signals than those transmitted by the sperm cells may program offspring (Robertson, 2007). As in blood, extracellular vesicles (EVs) are present in seminal fluid and carry miRNA. EVs are membrane-bound vesicles that carry and transfer molecules mediating intercellular communications (Cho et al., 2018). Recent studies indicated that they seem to play a role in gametogenesis, post-testicular sperm maturation, and fertilization and to contribute to embryo development (Machtinger et al., 2016). Seminal plasma is essential for sperm protection and maturation. It contains proteins that can modulate sperm functions through exosomes bound to spermatozoa surface (Samanta et al., 2018). Small extracellular vesicles (sEVs) of seminal fluid contain miRNAs that are sensitive to physiological conditions. Indeed, in azoospermic men, miRNA profiles of seminal plasma sEVs vary according the presence of spermatozoa obtained from the testicular biopsy (Barcelo et al., 2018). Epididymosomes, secreted from the epididymal stroma, are able to convey protein cargo to the sperm (Nixon et al., 2019). They play a critical role in post-testicular sperm maturation and can transfer miRNA to mature spermatozoa (Reilly et al., 2016). Furthermore, circulating EV-derived miRNAs have been identified as biomarker of early embryonic viability in cattle (Pohler et al., 2017), thereby suggesting their role in early embryonic development. Consequently, miRNAs present in the EVs, such as epididymosomes, and miRNAs free in seminal fluids may program offspring long-term health.

Concerning offspring phenotypes transmitted by paternal stress, the role of sncRNAs, and more particularly the role of miRNAs, has been highlighted. In addition, glucocorticoids, such as corticosterone, seem deeply involved in this phenomenon (Pang et al., 2017). Thus, Chan et al. have shown that the miRNAs present in the epididymosomes of the epididymis cauda are regulated by glucocorticoid receptors and play a crucial role in the transmission of paternal stress (Chan et al., 2018). These data suggest that the endocrine system may be involved in paternal programming. Other hormonal pathways may also be involved in the transmission of metabolic diseases.

## Perspectives

It is well known that paternal obesity or metabolic disorders may alter sperm parameters and ART outcomes (Campbell et al., 2015). There is now growing evidence that paternal obesity also programs a high susceptibility to develop metabolic syndrome in the offspring with potential further male infertility. In a model of HFD-fed mice, an alteration of sperm parameters has been reported in obese males (Bakos et al., 2011; Mitchell et al., 2011) with an alteration of both metabolic and reproductive health of the offspring. Male offspring presented altered sperm parameters, and female offspring oocytes presented reduced meiotic competence and altered mitochondrial membrane potential (Fullston et al., 2012).

Although they are rare in human, some interventional studies have shown a beneficial effect of weight loss in overweight or obese men on sperm quality or male fertility (Hakonsen et al., 2011; Faure et al., 2014; Lee et al., 2019). These obese fathers improved metabolic health when subjected to diet and/or physical activity, and this was also associated with an improvement of their sperm parameters (Palmer et al., 2012). This weight loss associated with physical activity had a beneficial effect on embryonic development, and the offspring showed an improvement in the several metabolic features. miRNA changes may be involved in this phenomenon. Indeed, a normalization of the sperm miRNA profile of the obese fathers subjected to physical intervention and an improvement of the expression of the pancreatic miRNA in the female offspring was observed (McPherson et al., 2015, 2017). In humans, no data are available for miRNA, whereas deep changes of methylation profile have been reported after bariatric surgery (Donkin et al., 2016).

Data assessing the impact of paternal exercise on offspring health are scarce and controversial, but the effect of physical exercise before conception seems beneficial, especially in case of paternal obesity. The model of fathers fed a standard diet and subjected to long-term physical exercise may be similar to models of fathers exposed to undernutrition programs (Murashov et al., 2016). Thus, in a paternal undernourished model, offspring presented small birth weight, associated with a catch-up growth and increased adiposity and dyslipidemia later on. In this model, supplementation with vitamins and antioxidants (vitamin C, vitamin E, folate, lycopene, zinc, selenium, and green tea extract) allowed normalization of sperm damages caused by undernutrition (increase in oxidative lesions and decrease in overall methylation). Interestingly, it also prevented deleterious effects in the offspring (McPherson et al., 2016).

Other environmental disorders or factors affecting paternal lifestyle may have deleterious consequences on offspring health, such as paternal smoking (Beal et al., 2017). Furthermore, several studies have highlighted a transmission of paternal trauma and stress to the offspring through miRNAs (Gapp et al., 2014; Fullston et al., 2017). Moreover, other outcomes than metabolic ones may also be challenged by paternal environment, suggesting a wide range of consequences for the offspring, like breast cancer risk in daughter (for example, Fontelles et al., 2016).

Thus, multiple environmental signals are able to induce epigenetic changes in the male gamete. Importantly, these modifications are environment-specific since they are able to induce in the respective progenies the phenotype of the father. Which molecular mechanism is involved in this specific modulation of sperm epigenome and/or sperm miRNAs? This remains an open question. To answer this question, it might be interesting to discriminate and identify the specific epigenetic modifications induced by each environment. This could be useful not only to better understand the general molecular mechanism involved in the process of epigenetic inheritance but also to identify specific stress markers. Whatever the molecular mechanism involved in the establishment of new spermatic epigenetic marks, it seems likely that these signals once transferred into the zygote are able to induce modifications, which can be transmitted throughout development. Which specific modifications are involved and how are they maintained? To tackle challenges arising from these questions, a global epigenomic analysis – i.e., ATAC-seq and DNA methylome analysis – on the modified embryos at a very early stage of development could be performed. This would lead us – on the long view – to identify loci epigenetically targeted by environmental signals.

## Conclusion

The periconceptional environment and lifestyle factors modify sperm epigenome. This alteration might be maintained in the zygote and throughout development, thereby leading to the inheritance of newly acquired pathologies. The role of sperm miRNA, not only as innovative markers of fertility issues but also as vectors involved in the inheritance of paternal diseases, appears to be crucial. Overweight and obesity seem to alter sperm miRNA profile, thereby leading to transmission of different miRNA profiles in zygote, with consequences on embryo development. In long term, metabolic disorders have been described in offspring F1 and F2, and other pathologies may originate from paternal environment.

In practice, this concept is important to know because it can help to predict and improve the periconceptional period, with a personalized management, and could be a powerful tool for infertility care. Indeed, paternal environment is too often neglected compared to the maternal environment. Identifying sperm miRNA profiles to predict risk of fertility alteration or noncommunicable disease inheritance is now required and needs further investigation, especially in randomized control trials in human. The impact of selected miRNA on IVF outcome, and the possibility to reverse or modify these miRNA profiles, requires our interest. Health professionals should pay more attention to the health of men of childbearing age.

## Author Contributions

CD, LK, SS, VG, and RL participated in the manuscript’s conception and design. CD, LK, and VG participated in drafting of the manuscript. RL participated in critical revision of the manuscript for intellectual content and supervised the study.

### Conflict of Interest Statement

The authors declare that the research was conducted in the absence of any commercial or financial relationships that could be construed as a potential conflict of interest.
